# Transformational leadership and project success: the mediating roles of team reflexivity and project team resilience

**DOI:** 10.3389/fpsyg.2025.1504108

**Published:** 2025-04-30

**Authors:** Huibin Han, Chihao Ma, Danning Yang, Weiwei Zhao

**Affiliations:** ^1^School of Economics and Management, Liaoning University of Technology, Jinzhou, Liaoning, China; ^2^School of Management, Liaoning Institute of Science and Engineering, Jinzhou, Liaoning, China

**Keywords:** transformational leadership, team reflexivity, project team resilience, project success, project management

## Abstract

Drawing from social cognitive theory, this study examines the mediating roles of team reflexivity and project team resilience in translating transformational leadership into project success. Data were collected from 261 project team members across various construction firms in China. The findings reveal that transformational leadership demonstrates a direct positive influence on project success. Moreover, transformational leadership significantly enhances both team reflexivity and project team resilience, which in turn contribute to project success. Furthermore, the results indicate that team reflexivity and project team resilience mediate the relationship between transformational leadership and project success. Based on these results, theoretical implications and practical recommendations are provided.

## 1 Introduction

The construction projects face increasingly dynamic and uncertain project environments, leading to persistent challenges for project success (Akinosho et al., 2020). Recent research has identified ineffective leadership in managing uncertainties as a primary contributor to project failures (Sanchez-Manzanares et al., 2020). Among various leadership approaches, transformational leadership has demonstrated particular promise in dynamic project environments (Wang et al., 2017).

Although previous studies have explored various mediators linking transformational leadership to project success at the individual level, scholars increasingly emphasize the need for a more comprehensive exploration of team-level mechanisms (Ahmad et al., 2022; Fareed and Su, 2022; Fareed et al., 2022). Given that teams have become the core operational units in project-based organizations (Lei et al., 2022), research has highlighted constructs such as team-building and teamwork quality as facilitators of leadership effectiveness in projects (Aga et al., 2016; Ali et al., 2021). However, there has been limited research on the mediating roles of adaptation-relevant team processes between transformational leadership and project success.

To address this gap, this study investigates the mediating roles of two team adaptation processes: team reflexivity and project team resilience. Unlike team-building and teamwork quality, which primarily enhance intra-team coordination, team reflexivity serves as a proactive adaptation process, enabling teams to engage in collective reflection before challenges arise (Liu et al., 2025). Conversely, project team resilience functions as a reactive adaptation activated when teams face challenges and adversity (Pavez et al., 2021). Where reflexivity may fail to anticipate certain challenges, resilience provides the necessary alternative for teams to respond effectively to unforeseen circumstances. By integrating both proactive and reactive adaptation processes, this study offers a more holistic perspective on how transformational leadership influences project success.

Social Cognitive Theory (SCT) suggests that team members regulate their behaviors and attitudes through observational learning and social modeling (Bandura, 1999). Within this framework, transformational leaders serve as social models, shaping how team members perceive challenges and respond to uncertainties. In this research, we contend that transformational leaders cultivate a team environment that encourages adaptive learning and problem-solving by showing intellectual stimulation (Avolio et al., 2004). In this atmosphere, team reflexivity emerges as a cognitive adaptation mechanism that enables team members to critically evaluate problems and continuously refine their approaches. Furthermore, the value of team reflexivity for team performance has been well documented (Lei et al., 2022). Therefore, this study aims to uncover the mediating role of team reflexivity between transformational leadership and project success.

In addition, Transformational leaders foster a culture of continuous learning, encouraging teams to embrace setbacks as opportunities for growth and improvement (Sahin and Bilir, 2024). Through observational learning mechanisms, team members internalize leaders' coping strategies when confronting adversities. Project team resilience operates as a behavioral adaptation process that allows teams to recover from operational failures and sustain performance efficacy under volatile conditions (Pavez et al., 2021). Moreover, the relationship between team resilience and positive team outcomes is well established (Meneghel et al., 2016). Consequently, this study also seeks to explore the mediating role of project team resilience between transformational leadership and project success.

In sum, this study investigates how transformational leadership influences project success by examining the mediating roles of team reflexivity and project team resilience as possible adaptation processes. It makes two primary contributions to the literature. First, it establishes a dual-pathway adaptation mediation, where team reflexivity (proactive adaptation) and project team resilience (reactive adaptation) function as critical mechanisms linking transformational leadership to project success. Second, it deepens the understanding of how transformational leadership fosters project success by analyzing the dynamic interplay between leadership behaviors, team reflexivity, and team resilience. This perspective shifts the focus from identifying leadership traits to exploring how leaders enable teams to adapt and reconfigure their competencies in dynamic project environments.

## 2 Theoretical background and literature review

### 2.1 Social cognitive theory

SCT, developed by Bandura (1999), provides a comprehensive framework for understanding team behaviors through the concept of triadic reciprocal determinism, which describes the dynamic interaction between personal factors, behavior, and the environment. This interaction is fundamental in shaping both individual and team actions within a given context. According to SCT, behaviors are not solely influenced by one factor, but rather emerge from the ongoing, reciprocal interplay between these three elements.

Within this framework, there are two key learning mechanisms that explain how individuals acquire and refine behaviors. The first is observational learning, also known as modeling, which involves learning by observing the behaviors of others, particularly those who are regarded as role models. The second mechanism is vicarious learning, which occurs when individuals learn by observing the outcomes of others' actions. By witnessing the consequences—both positive and negative—of others' behaviors, team members adjust their own actions accordingly, often avoiding mistakes and replicating successful strategies (Wood and Bandura, 1989).

SCT also highlights two reinforcing mechanisms that influence behavior and development. Self-regulation is a crucial process by which individuals manage their emotions, thoughts, and actions in pursuit of long-term goals. Additionally, SCT emphasizes self-reinforcing processes, where individuals' actions create a cycle of positive reinforcement. When a behavior leads to successful outcomes, it reinforces that behavior, creating a virtuous cycle that promotes continued engagement and improvement (Lin et al., 2020).

### 2.2 Transformational leadership

Transformational leadership is a leadership style that motivates members to transform their beliefs and values to increase their performance over self-interest and prioritize the organization's needs (Avolio et al., 1999). The transformational leadership theory, which Burns proposed in 1978, envisions as the ideal relationship between political leaders and supporters. Bass developed the theory into the research of organizations, stating that transformational leadership could encourage members to sacrifice their personal interests by allowing them to recognize the importance of their tasks (Bass, 1999). Based on this research, a lot of effort has been made by numerous scholars to study transformational leaders' characteristics and their positive effects on organizational outcomes. Since then, transformational leadership has acquired wide acceptance in leadership studies and project management (Antonakis and House, 2013).

In project-based organizations, transformational leadership demonstrates significant advantages (Aga et al., 2016). A comprehensive meta-analysis conducted by Hoch et al. (2018) reveals that transformational leadership demonstrates superior efficacy compared to other moral value-based leadership styles in elucidating followers' behaviors, attitudes, and perceptions within relational contexts. Research indicates that in project-based organizations, transformational leadership positively correlates with higher performance, team innovation, and job satisfaction (Zhu et al., 2013; Al-edenat, 2018; Le and Lei, 2019). In addition, the advantages of transformational leadership become more pronounced when facing highly dynamic and complex project environments, better promoting organizational adaptability and long-term success (Han et al., 2024). Notably, the effectiveness of transformational leadership appears to be more evident in developing countries, including China, than in Western countries (Crede et al., 2019).

This study employed a short measurement scale called Global Transformational Leadership scale (GTL) developed by Carless et al. (2000) to measure transformational leadership. As the primary aim of this study is to examine the overall impact of transformational leadership on other variables, rather than the effects of its individual components. The GTL's global assessment of transformational leadership is particularly suited to this purpose. As the GTL “provides a broad assessment of transformational leadership” (Carless et al., 2000, p. 402), encompassing key aspects such as vision, staff development, support, empowerment, innovation, leading by example, and charisma in a single construct. Moreover, the GTL has been successfully employed in various empirical studies across different cultural contexts (Alwali and Alwali, 2022; Kloutsiniotis et al., 2022; Shaikh et al., 2023). Thus, the GTL, alignment with recent theoretical critiques, practical brevity, and robust psychometric properties make it an ideal instrument for this study's objectives.

### 2.3 Team reflexivity

Team reflexivity has emerged as a crucial concept in organizational behavior and team performance studies, particularly in the context of project management. Team reflexivity is a proactive and adaptive process in which team members actively reflect on and discuss their objectives, strategies, and workflows, making adjustments based on current circumstances (West and Anderson, 1996). It involves a conscious effort to evaluate, question, and potentially adjust how the group operates, with the aim of boosting performance and realizing intended results. The practice of team reflexivity facilitates a valuable exchange of ideas among team members. Through this collaborative reflection, teams can uncover more efficient work methods and explore innovative approaches to tasks. Importantly, this process encourages thoughtful improvement rather than resorting to shortcuts merely to complete assignments quickly (Ren et al., 2021).

### 2.4 Project team resilience

Unlike general team resilience which is defined as “the capacity of a team to withstand and overcome stressors in a manner that enables sustained performance” (Alliger et al., 2015, p. 177), project team resilience is characterized by its time-bound nature, unique project-specific challenges, and dynamic team composition (Varajão et al., 2021). Specifically, project teams operate within defined time frames, often with strict deadlines and milestones (Scott-Young and Samson, 2008). Additionally, project teams frequently experience changes in membership and leadership throughout the project lifecycle (Kloppenborg and Petrick, 1999). While team resilience and project team resilience share foundational elements, the latter represents a specialized construct that addresses the unique demands of project-based work environments. This study adopts the definition proposed by Pavez et al. (2021, p. 699), which describes project team resilience as “the capacity to anticipate, contain, and recover from adversity or failure induced by the uncertainty and complexity of a project environment”. This definition is particularly appropriate as it captures the reactive adaptation component of resilience within the specific context of project management, evident in the capacity to contain and recover from challenges as they arise.

### 2.5 Project success

Despite ongoing research since the 1970s (Ika, 2009), the definition of project success remains a subject of debate (Jugdev and Müller, 2005). In the 1990s, project success was commonly defined by the “iron triangle”—constrained by time, quality, and cost (Atkinson, 1999). Pinto and Mantel (1990) proposed a systemic, three-dimensional approach to define project success, including project efficiency, perceived quality, and client satisfaction. According to the Project Management Institute (PMI), project success has evolved over the past two decades to encompass a balance among cost, time, quality, scope, and stakeholder satisfaction (Aga et al., 2016). Similarly, Raziq et al. (2018) suggest that project success includes meeting scope, time, cost, and quality objectives, as well as satisfying customers and stakeholders and achieving the project's objectives.

Over time, the concept of project success has expanded to include additional dimensions and factors. Although its definition has not yet reached a wider consensus, project success has evolved from the traditional focus on the iron triangle including cost, time, and quality into a broader understanding of success that considers multiple perspectives (Pollack et al., 2018). Thus, rather than evaluating a single aspect of performance, using composite measures such as performance, efficiency, effectiveness, impact, and sustainability for assessing project success may serve as more comprehensive indicators for overall team performance (Aga et al., 2016).

## 3 Hypothesis development

### 3.1 Transformational leadership and project success

Transformational leaders develop and communicate a compelling vision through systematic and consistent messaging that establishes clear direction and purpose (Bush, 2018). This vision-setting process creates favorable environmental conditions that positively influence team members' behaviors, strengthen their self-efficacy beliefs, and shape their outcome expectations. Through deliberate modeling of desired behaviors and explicit articulation of core values, transformational leaders facilitate the alignment between individual efforts and organizational objectives while simultaneously fostering a deeper sense of purpose in team members' work (Carless et al., 2000). Furthermore, transformational leadership elevates performance standards through intellectual stimulation, particularly in goal-setting processes (Avolio et al., 1999). The establishment of clear, well-defined, and appropriately challenging goals has been demonstrated to enhance both motivation and performance outcomes (Locke and Latham, 2002). By encouraging team members to establish and pursue ambitious standards for themselves, transformational leaders cultivate a growth mindset within the team. This approach nurtures a collective sense of responsibility, which consequently leads to improvements in both the efficiency and effectiveness of project execution. Therefore, the following hypothesis is proposed:

**Hypothesis 1 (H1):** Transformational leadership positively impacts project success.

### 3.2 The mediating role of team reflexivity between transformational leadership and project success

Transformational leadership fosters team reflexivity through modeling reflective behaviors and reinforcing these behaviors via observational learning and feedback. Transformational leaders act as role models by demonstrating reflective practices themselves (Schippers et al., 2008), which team members then observe and internalize. These modeled behaviors create opportunities for observation learning, where team members replicate the reflective actions. This process is further strengthened by feedback from the leader, reinforcing the behavior and encouraging a continuous cycle of reflection. In this way, observational learning strengthens the commitment to reflection as a team norm. Furthermore, transformational leaders enhance team reflexivity by promoting self-regulation through collective monitoring and goal-setting. Transformational leaders facilitate this process by guiding teams in setting shared goals and evaluating their collective progress (Chai et al., 2017). As team members monitor their progress toward these goals and adjust their strategies accordingly, they engage in self-regulatory behaviors that are central to team reflexivity. This continuous evaluation and adaptation process is reinforced by feedback from leaders, which further promotes reflection and improvement. By fostering observational learning and self-regulation, transformational leadership creates an environment where reflexivity is central to team behavior. Thus, the following hypothesis is suggested:

**Hypothesis 2a (H2a):** Transformational leadership positively impacts team reflexivity.

Team reflexivity significantly enhances team performance through its facilitation of organizational learning and comprehensive knowledge sharing within teams. When team members engage in reflexive practices, they create opportunities for vicarious learning, allowing colleagues to benefit from shared experiences without direct exposure (Bandura, 1999). The combination of diverse viewpoints from different disciplines enriches the learning environment (Bell and Kozlowski, 2002), leading to more comprehensive problem analysis and innovative solutions. This multi-faceted approach to learning and problem-solving through reflexivity ultimately strengthens project outcomes. In addition, when teams engage in reflexive practices, they create structured chances for members to articulate their unique insights, experiences, and expertise (Schippers et al., 2015), making both tacit and explicit knowledge accessible to the entire team (Wang et al., 2021). This process is particularly effective because reflexivity creates a psychologically safe environment. In this context, team members feel comfortable sharing not only successes but also failures and concerns (Edmondson, 1999), enabling the exchange of sensitive yet valuable knowledge that might otherwise remain hidden. Through reflexive discussions, teams engage in collective sense-making processes that transform raw information into actionable knowledge (Marks et al., 2001). Moreover, reflexivity promotes the practical application of shared knowledge by helping teams identify relevant experiences and adapt them to current project challenges (Hoegl and Parboteeah, 2006). The combination of organizational learning climate and comprehensive knowledge sharing through team reflexivity ultimately creates a robust foundation for improving project outcomes. Therefore, the hypothesis is proposed:

**Hypothesis 2b (H2b):** Team reflexivity positively impacts project success.

Transformational leadership, characterized by behaviors that support, motivate, and innovate (Carless et al., 2000), fosters an environment conducive to reflection, learning, and adaptation. By encouraging open communication, transformational leaders facilitate teams' reflexive actions and potentially adjust their operations in alignment with team goals and project demands (Schippers et al., 2008). The regular engagement of team members in reflexive practices, facilitates idea exchange and uncovers more efficient work methods and innovative task approaches (Ren et al., 2021). These behaviors activate reciprocal determinism, reflecting a dynamic interaction between cognitive processes (critical thinking and reflection) and behavioral adjustments (adopting new strategies) (Bandura, 1999). Through this reciprocal process, team members develop enhanced collective capabilities for assessment and response. As a result, reflexive teams demonstrate enhanced adaptation to changing circumstances, early problem identification, and informed decision-making capabilities, all of which contribute to higher project success rates (Giezen et al., 2015; Ika and Donnelly, 2017). The active engagement in reflexivity encourages teams to pursue thoughtful improvements rather than resorting to shortcuts for quick task completion. In this context, teams regulate their behavior through reflection and feedback, leading to goal attainment and enhanced performance (Schippers et al., 2015). Thus, by fostering team reflexivity, transformational leadership creates a self-reinforcing cycle of continuous improvement and adaptation, ultimately enhancing project success. Thus, the hypothesis is suggested:

**Hypothesis 2c (H2c):** Team reflexivity positively mediates the relationship between transformational leadership and project success.

### 3.3 The mediating role of project team resilience between transformational leadership and project success

Transformational leadership positively impacts project team resilience by developing team members' skills and acting as resilient models. Transformational leaders prioritize the development of team members by providing training, mentoring, and growth opportunities, which equip individuals with the necessary skills to handle adversity (Avolio et al., 1999). This focus on skill-building not only enhances individual competencies but also strengthens collective resilience. By empowering team members through skill development, transformational leaders contribute to building a resilient team capable of overcoming uncertainty. Additionally, transformational leaders foster a culture where risk-taking and innovation are seen as positive responses to adversity (Mokhber et al., 2018). In this environment, team members observe how their leader handles challenges and are encouraged to adopt similar strategies (Wood and Bandura, 1989). Leaders model how setbacks can be viewed as learning experiences, thereby promoting a growth mindset within the team. This process of modeling behaviors, reinforced by feedback and social support, encourages team members to approach difficulties proactively rather than defensively. Thus, the hypothesis is suggested:

**Hypothesis 3a (H3a):** Transformational leadership positively impacts project team resilience.

Project team resilience positively impacts project success by enabling teams to navigate challenges and maintain performance stability. In construction projects, which are complex and involve multiple stakeholders, team resilience is crucial for maintaining project momentum in the face of unforeseen challenges. A resilient project team is better equipped to handle the dynamic nature of construction projects. As Jiang et al. (2024) emphasize, robust project team resilience enables teams to effectively adapt to challenges and stay on track toward achieving project goals. Thus, project team resilience directly contributes to project success by fostering the capacity to overcome obstacles and sustain performance. In addition, when a project team encounters unexpected challenges, its ability to contain and recover from these difficulties is vital for maintaining project momentum. Watson et al. (2001) suggests that overcoming adversity boosts collective efficacy beliefs, which increases team persistence in the face of future obstacles. Additionally, successful recovery serves as a learning opportunity, where team members observe and replicate problem-solving behaviors through observational learning. This cyclical process of recovery and learning fosters resilience and enhances the team's ability to solve problems effectively. Therefore, the hypothesis is suggested:

**Hypothesis 3b (H3b):** Project team resilience positively impacts project success.

Transformational leaders act as models, exhibiting behaviors and attitudes that team members can observe and emulate (Khan and Khan, 2022). This observational learning process is particularly efficacious due to the reverence and esteem typically accorded to transformational leaders (Arnold, 2017). By fostering innovative thinking and encouraging cognitive diversity (Kahai et al., 2013; Le and Lei, 2019), transformational leaders strengthen the team's ability to anticipate and adapt to challenges. Furthermore, by enhancing team members' self-efficacy and confidence (Lu and Li, 2021), transformational leaders reinforce the collective belief in the team's capability to overcome adversity. As a result, the team develops greater resilience, enabling it to effectively manage stressors and maintain stability in complex environments. Resilient teams demonstrate the ability to contain and recover from setbacks, maintaining performance stability in dynamic project environments (Alliger et al., 2015). Their capacity to withstand and overcome stressors enables the sustainment of long-term project performance (Stoverink et al., 2020). By fostering resilience through their leadership behaviors, transformational leaders create an adaptive team culture, equipping members to better manage the complexities inherent in project environments. Ultimately, this cultivated resilience serves as a crucial driver of project success. Consequently, the following hypothesis is proposed:

**Hypothesis 3c (H3c):** Project team resilience positively mediates the relationship between transformational leadership and project success.

**Figure 1:**
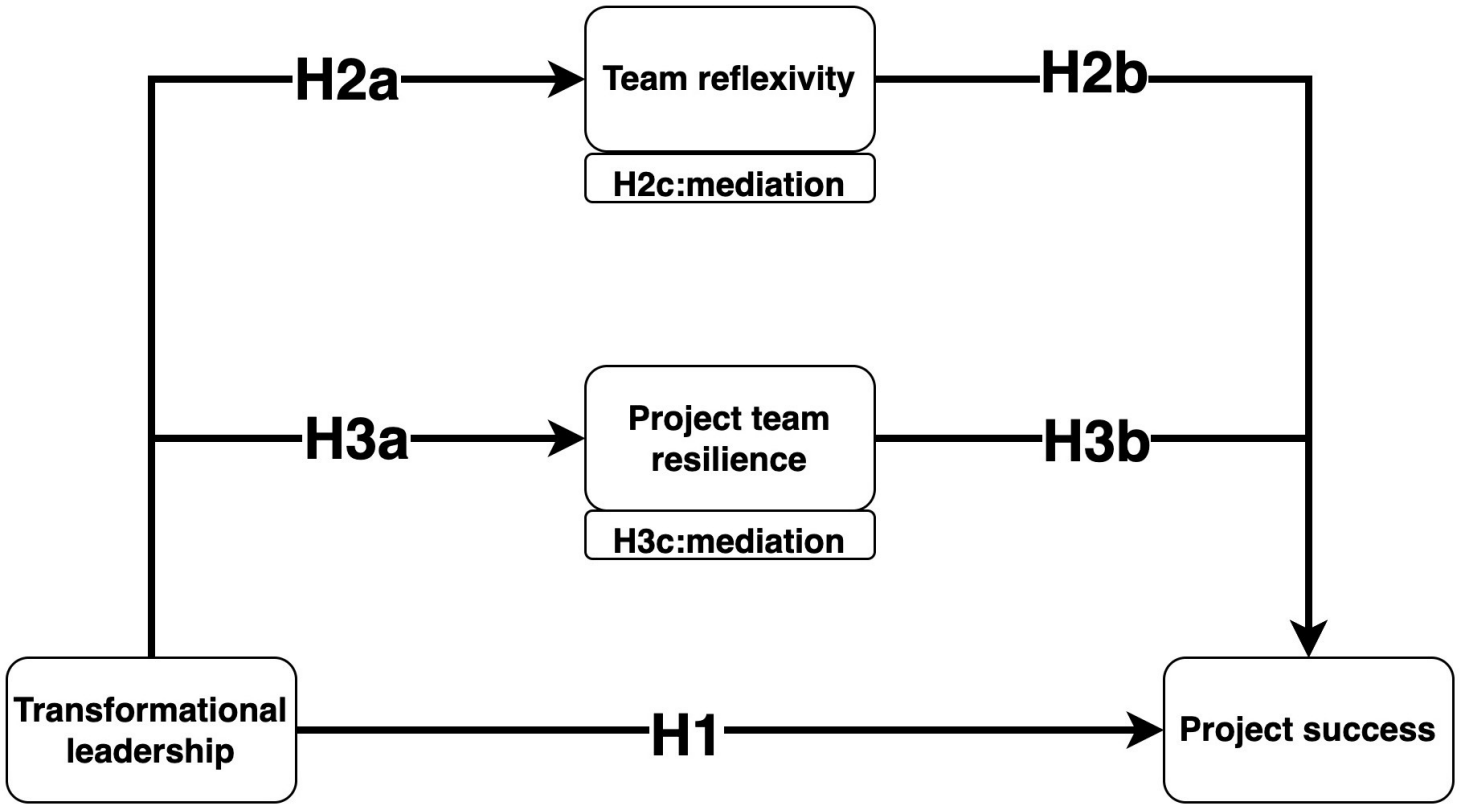
Conceptual model.

In summary, this study enriches the literature on project management by examining the mediating roles of team reflexivity and project team resilience between transformational leadership and project success. The proposed model is depicted in Figure 1.

## 4 Methods

### 4.1 Sample and procedure

The sample for this study comprised 261 respondents actively engaged in various construction projects throughout China. Data collection was strategically conducted through alumni networks specializing in engineering management and construction cost from three prominent universities in Liaoning Province in China. This approach ensured access to a well-informed and relevant participant pool, enhancing the reliability and applicability of the study's findings within the construction industry context.

The questionnaire was initially developed based on previous relevant studies, with items translated from English into Chinese. Subsequently, two professors specializing in engineering management meticulously reviewed the questionnaire to identify and correct any inconsistencies. A pilot test was then conducted with a small sample of 10 respondents (quantity surveyors), and the questionnaire was further revised based on their feedback before being distributed to the target participants.

The questionnaire was structured into three distinct sections. The first section served as an introduction, outlining the survey's objectives, assuring respondents of the confidentiality of their answers, and providing detailed instructions for completion. The second section was dedicated to collecting demographic information, including respondents' age, years of experience, and educational background. The final section encompassed the variable measurement scales, containing questions designed to assess the key constructs central to the study.

The questionnaire was distributed to a targeted sample of professionals in the construction industry using Wenjuanxing, a popular online survey platform in China. The initial sample consisted of 20 qualified respondents (quantity surveyors) who were selected from the alumni networks specializing in engineering management and construction cost from three prominent universities in Liaoning Province, China. These respondents met the following inclusion criteria: (1) having at least 2 years of project management experience; (2) being currently employed in the construction industry; and (3) holding a professional qualification as a quantity surveyor.

To expand the sample size, these initial participants were asked to forward the questionnaire to other similarly qualified individuals within their professional circles. This snowball sampling technique was employed to reach a broader audience while maintaining the relevance of the sample to the study context. Data collection took place from December 2023 to March 2024, coinciding with the winter engineering break in northern China. During this period, quantity surveyors typically have a lighter workload, which allowed for more focused and higher-quality responses to the questionnaire. This timing was strategically chosen to ensure the reliability and validity of the data collected.

Over the four-month data collection period, a total of 307 questionnaires were collected. Of these, 261 were deemed valid for analysis based on the following exclusion criteria: (1) responses that were identical across all questions, indicating a lack of genuine engagement with the survey; and (2) illogical responses, such as individuals reporting an age of 26 with over 13 years of experience. These criteria were applied to ensure the integrity and credibility of the dataset. Table 1 provides the demographic details of the remaining participants.

**Table 1:** Demographic characteristics of the sample.

**Features**	**Category**	**Frequency**	**Percentage**
Gender	Male	136	52.11%
	Female	125	47.89%
Age (years)	20–30	46	17.62%
	30–40	91	34.87%
	40–50	80	30.65%
	50–60	44	16.86%
Education	Below undergraduate	155	59.38%
	Undergraduate	85	32.57%
	Master and above	21	8.05%
Experience (years)	< 3	1	0.39%
	3–5	31	11.88%
	5–10	49	18.77%
	10–15	39	14.94%
	>15	141	54.02%
Duration (years)	< 5	100	38.31%
	5–10	72	27.59%
	10–15	43	16.48%
	>15	46	17.62%

### 4.2 Measurement

Participants, unless stated otherwise, responded using a five-point Likert scale, where 1 represented “Strongly Disagree” and 5 represented “Strongly Agree.” The following section outlines the measurements used in this study:

Transformational leadership was assessed using the Global Transformational Leadership scale (GTL) from Carless et al. (2000). An example item is: “My project manager communicates a clear and positive vision of the future.” (Cronbach's α = 0.899).

Project success was measured by nine items from Aga et al. (2016). An example item is: “The project was completed on time.” (Cronbach's α = 0.907).

Project team resilience was measured using four items adapted from Pavez et al. (2021). A sample item is: “In my team, we cope well with the conflicts we experience at work.” (Cronbach's α = 0.894).

Team reflexivity was assessed using four items from Elbanna (2015). A sample item is: “Our team's responsiveness to changing organizational conditions is timely.” (Cronbach's α = 0.881).

Demographic factors such as gender, experience, age, and educational background were considered. Additionally, team size and project duration were included in the analysis (Aga et al., 2016).

## 5 Analysis and results

RStudio Version 2024 was utilized to analyze the data. Confirmatory factor analysis (CFA) was implemented to authenticate the measurement patterns denoting the variables within the overarching structural equation model. Furthermore, SEM techniques were utilized to evaluate the proposed model and examine the postulated assumptions. In addition, the application of bootstrap methods provides a robust mechanism for assessing the hypothesized relationships, thereby ensuring a comprehensive and rigorous examination of the assumptions.

### 5.1 Reliability and validity

As shown in Table 2, the Cronbach's Alpha and Composite Reliability values for constructs are all above the recommended thresholds (0.7), indicating high internal consistency (Vaske et al., 2017). The Average Variance Extracted (AVE) values exceed 0.50 for all constructs, supporting good convergent validity. Discriminant validity is confirmed as the square root of the AVE for each construct is higher than its correlations with other constructs. Additionally, as shown in Table 3, the baseline model, which reflects the original factor structure, shows excellent fit indices (χ^2^/df = 1.035, RMSEA = 0.012, CFI = 0.998, TLI = 0.997, SRMR = 0.036), further validating the measurement model and ensuring that the constructs are distinct and accurately measured (Dogan and Özdamar, 2017).

**Table 2:** Pearson's correlation matrix, reliability, and validity.

**Variable**	**Item loading**	**Cronbach's α**	**CR**	**AVE**	**TSL**	**TR**	**TI**	**PS**
**TSL**	0.643~0.859	0.899	0.901	0.570	(0.755)			
**PTR**	0.794~0.873	0.894	0.896	0.686	0.425^**^	(0.828)		
**TR**	0.711~0.892	0.881	0.892	0.682	0.433^**^	0.469^**^	(0.826)	
**PS**	0.594~0.851	0.907	0.911	0.542	0.468^**^	0.515^**^	0.567^**^	(0.736)

TSL, transformational leadership; PTR, project teamresilience; TR, teamreflexivity; PS, project success; ^**^ means the correlation is significant at the 0.01 level (2-tailed); The values in parentheses are the square root of AVE.

**Table 3:** Comparison of measurement models.

**Model**	**χ2**	**df**	**χ2/df**	**RMSEA**	**CFI**	**TLI**	**SRMR**
Baseline model	254.723	246.000	1.035	0.012	0.998	0.997	0.036
Three-factor model	672.691	249.000	2.702	0.081	0.883	0.870	0.060
Two-factor model	1186.429	251.000	4.727	0.119	0.741	0.715	0.100
One-factor model	1591.188	252.000	6.314	0.143	0.629	0.593	0.114

Three-factor model: PTR and TR merged into one factor. Two-factor model: TSL, PTR, and TR merged into one factor. One-factor model: TSL, PTR, TR, and PS merged into one factor.

### 5.2 Common method bias

Two approaches were employed to evaluate the common method bias. First, the initial principal component explained 40% of the total variance, which is below the 50% threshold typically associated with significant common method bias (Podsakoff et al., 2003). Second, as shown in Table 3, the baseline model, which aligns with the original factor structure, demonstrates excellent fit indices (χ^2^/df = 1.035, RMSEA = 0.012, CFI = 0.998, TLI = 0.997, SRMR = 0.036). In contrast, the fit significantly worsens in the three-factor, two-factor, and one-factor models, with the one-factor model showing the poorest fit (χ^2^/df = 6.314, RMSEA = 0.143, CFI = 0.629, TLI = 0.593, SRMR = 0.114). This decline in fit suggests that common method bias is unlikely (Podsakoff et al., 2003; Guo et al., 2016). The collective results indicate that common method bias may not pose a significant concern.

### 5.3 Hypothesis testing

**Figure 2:**
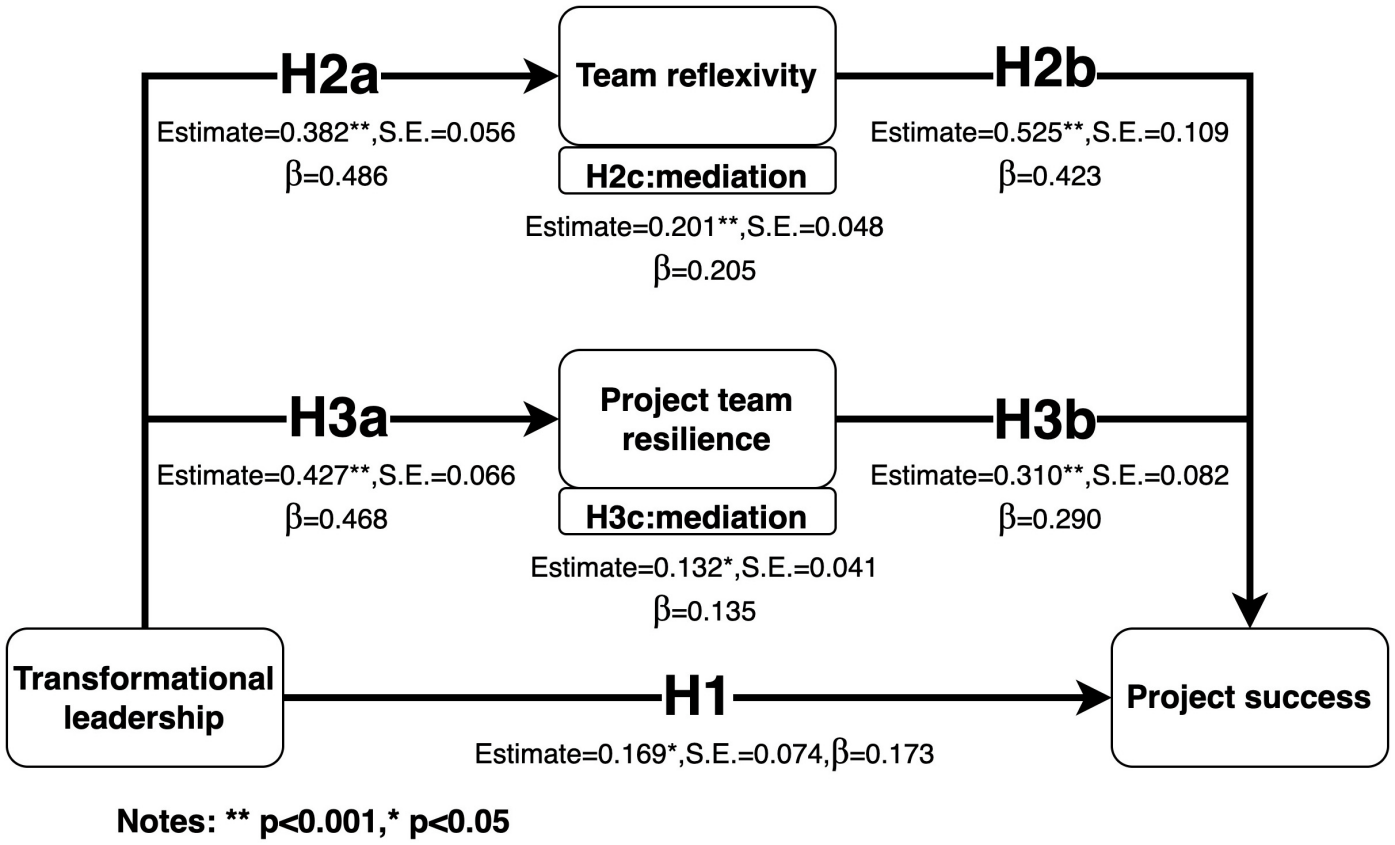
Mediation model.

Table 4 and Figure 2 present the path coefficients and results for hypothesis testing, detailing whether each hypothesis is supported:

**Table 4:** Path coefficients and hypothesis testing results.

**ID**	**Hypotheses**	**Estimate**	**β**	**S.E**.	***p*-Value**	**95% bias–corrected confidence interval**	**Results**
**Direct effects**							
**H1**	**TSL-PS**	0.169^*^	0.173	0.074	0.021	[0.022, 0.315]	Positive
**H2a**	**TSL-TR**	0.382^**^	0.486	0.056	0.000	[0.277, 0.493]	Positive
**H2b**	**TR-PS**	0.525^**^	0.423	0.109	0.000	[0.324, 0.750]	Positive
**H3a**	**TSL-PTR**	0.427^**^	0.468	0.066	0.000	[0.304, 0.560]	Positive
**H3b**	**PTR-PS**	0.310^**^	0.290	0.082	0.000	[0.157, 0.478]	Positive
**Mediating effects**							
**H2c**	**TSL-TR-PS**	0.201^**^	0.205	0.048	0.000	[0.117,0.304]	Positive
**H3c**	**TSL-PTR-PS**	0.132^*^	0.135	0.041	0.001	[0.063,0.221]	Positive

^**^p < 0.001; ^*^p < 0.05.

H1: Transformational leadership has a positive effect on project success with an estimate of β = 0.173 (*p* = 0.021). This effect is statistically significant, indicating that transformational leadership positively influences project success, thus supporting Hypothesis 1.

H2a: Transformational leadership significantly impacts team reflexivity with an estimate of β = 0.486 (*p* < 0.001). The result is highly significant, showing that transformational leadership positively affects team reflexivity, thereby supporting Hypothesis 2a.

H2b: Team reflexivity has a positive effect on project success with an estimate of β = 0.423 (*p* < 0.001). This effect is statistically significant, suggesting that team reflexivity positively influences project success, supporting Hypothesis 2b.

H3a: Transformational leadership significantly impacts project team resilience with an estimate of β = 0.468 (*p* < 0.001). The result confirms that transformational leadership positively affects project team resilience, thus supporting Hypothesis 3a.

H3b: Project team resilience has a significant positive effect on project success with an estimate of β = 0.290 (*p* < 0.001). This effect is highly significant, indicating that project team resilience positively influences project success, thereby supporting Hypothesis 3b.

H2c: Team reflexivity mediates the relationship between transformational leadership and project success with an estimate of β = 0.205 (*p* < 0.001) and a 95% confidence interval (CI) of [0.117, 0.304]. The confidence interval does not include zero, confirming that team reflexivity significantly mediates the relationship between transformational leadership and project success, thus supporting Hypothesis 2c.

H3c: Project team resilience mediates the relationship between transformational leadership and project success with an estimate of β = 0.135 (*p* = 0.001) and a 95% CI of [0.063, 0.221]. The confidence interval does not include zero, indicating that project team resilience significantly mediates the relationship between transformational leadership and project success, thus supporting Hypothesis 3c.

## 6 Discussion

The present study, grounded in SCT, investigated the direct and indirect relationships between transformational leadership and project success. As hypothesized, transformational leadership was found to have a significant impact on project success. This finding aligns with previous research conducted across various sectors and countries, including development projects in Ethiopia (Aga et al., 2016), telecom intensive companies in Pakistan (Zaman et al., 2019), public projects in Pakistan (Fareed and Su, 2022), and national public projects in Pakistan (Fareed et al., 2023). The results corroborate these insights, specifically in the context of China's construction industry, underscoring the pivotal role of transformational leadership in enhancing project success.

Secondly, this study reveals a positive relationship between team reflexivity and project success. This finding confirms that team reflexivity positively influences team performance but in construction project teams (Liu et al., 2025). The positive impact of team reflexivity on project success is particularly noteworthy given the heterogeneous nature of project teams. Project teams typically comprise members from diverse professional backgrounds, disciplines, and expertise levels. The heterogeneity aligns with the concept of heterogeneous learning orientation (Pieterse et al., 2011). Due to this heterogeneity, team reflexivity facilitates the sharing of experiences. This process allows team members to learn from each other's successes and failures. Moreover, reflective practices bring together varied viewpoints from team members, leading to more comprehensive problem analysis and innovative solutions. These mechanisms contribute to the development of more effective project management practices.

Thirdly, this study reveals a positive correlation between project team resilience and project success. This finding aligns with previous research on team resilience and team performance, highlighting the crucial role of resilience in teams. The importance of team resilience has been demonstrated across various domains. For instance, Meneghel et al. (2016) found that team resilience is related to both in-role and extra-role performance. Similarly, Z. Wang et al. (2023) corroborated these findings in the context of nursing teams, further emphasizing the universal impact of team resilience on performance. In the specific context of project management, our findings underscore the critical role of a team's ability to anticipate, adapt to, and recover from challenges. Project environments are inherently characterized by uncertainty, distinguishing them from more stable organizational contexts. This uncertainty manifests in various forms, such as changing stakeholder requirements, resource constraints, and unforeseen technical issues (Crawford et al., 2013). This finding underscores the critical importance of fostering resilience within project teams. By cultivating this attribute, project-based organizations can better equip their teams to navigate the inherent uncertainties of project environments.

Fourthly, perhaps more importantly, this study demonstrated that team reflexivity and project team resilience play mediating roles between transformational leadership and project success. This finding aligns with previous research on transformational leadership. For instance, Aga et al. (2016) found that team-building mediates the relationship between transformational leadership and project success in development projects. Similarly, Ali et al. (2021) demonstrated a serial mediation effect of team-building and teamwork quality in IT projects. Despite various mediators being identified, this is the first study explicitly identifying the mediating roles of team reflexivity and project team resilience, in the relationship between transformational leadership and project success.

### 6.1 Theoretical implication

This study makes several contributions to the existing body of knowledge in project management. Firstly, this study extends the application of SCT to the field of project management in the construction industry. Based on SCT, this study provides a theoretical framework for understanding the mechanisms through which transformational leadership influences project outcomes.

Secondly, by demonstrating the positive correlation between project team resilience and project success, this study extends the application of resilience theory to project management. It provides theoretical grounding for understanding how team resilience contributes to navigating the inherent uncertainties in project environments, building on previous work in other domains (Meneghel et al., 2016; Jiang et al., 2024). This expanded theoretical perspective on team resilience in project management. It underscores the importance of fostering resilience as a key team capability in the increasingly complex and uncertain landscape of modern project management.

Thirdly, this study makes a significant theoretical contribution by establishing a dual-pathway mediation model that elucidates the mechanisms through which transformational leadership influences project success. Specifically, our findings demonstrate that team reflexivity (as a proactive adaptation mechanism) and project team resilience (as a reactive adaptation mechanism) simultaneously function as mediating variables between transformational leadership and project outcomes. This finding extends the existing literature by not only illuminating how transformational leadership enhances project performance through facilitating team reflection, but also by clarifying how such leadership builds team capacity to respond effectively to challenges and recover from setbacks. This dual-pathway model provides a more comprehensive theoretical framework for understanding how leadership behaviors translate into project performance through distinct adaptive mechanisms.

### 6.2 Practical implication

Based on the findings, several practical implications can be identified for project management. As shown in Figure 2, despite having a lower β (0.173) compared to team reflexivity, transformational leadership has a direct and significant impact on project success. Moreover, transformational leadership indirectly influences project success by enhancing both team reflexivity (with a mediation β of 0.205) and project team resilience (with a mediation β of 0.135). This dual effect underscores the need for organizations to invest in the development of transformational leadership skills among project managers. It's suggested to provide leadership training programs that focus on project visions, empowerment, and participative decision-making. Furthermore, when applying transformational leadership in the Chinese context, it is essential to localize the leadership style by incorporating cultural values such as Confucian “benevolence”. This approach could align leadership practices with local cultural expectations and foster a more harmonious project environment. By integrating these cultural aspects, project managers can enhance their leadership effectiveness and contribute to the overall project success in China.

Secondly, leveraging SCT's concept of vicarious learning and integrating it with team reflexivity can significantly enhance project team management capabilities. As evidenced by the highest β (0.423) in Figure 2, team reflexivity emerges as the most critical factor influencing project success. By systematically studying and reflecting on the successes and failures of other projects, teams can gain valuable insights without directly experiencing every situation. This process involves learning initiatives from other similar projects and engaging in deep reflexive discussions. Teams can then translate these insights into actionable strategies and role-playing exercises. This comprehensive approach allows project teams to accelerate their learning process and develop more effective management practices. By combining vicarious learning with reflexive practices, teams can navigate the complexities of project management more adeptly.

Thirdly, enhancing project capabilities requires a dual-pronged approach that balances the development of project team resilience with the standardization of core processes. On one side, fostering project team resilience is crucial in today's dynamic project environments. Resilient teams involve cultivating a mindset of flexibility and developing problem-solving skills across the team. On the other side, solidifying standard processes provides a stable foundation for project management. By establishing and refining core procedures, teams can operate more efficiently and reduce errors. By simultaneously strengthening project team resilience and solidifying core processes, organizations can create a robust yet flexible project management environment to foster project success.

### 6.3 Limitations

The present study has several limitations. Firstly, it is important to acknowledge the limitations of the generalizability. The research was conducted primarily in Eastern cultures with a collectivist orientation, particularly in China and other Asian countries. This cultural context may influence the effectiveness and perception of transformational leadership, as well as its impact on project success. Collectivist cultures tend to emphasize group harmony, respect for authority, and long-term relationships (Triandis, 2001), which may amplify the effects of transformational leadership. In contrast, Western cultures, which are generally more individualistic, may respond differently to transformational leadership styles. Future research is suggested to explore moderating variables, such as team diversity, project complexity, or cultural factors, to deepen the study's implications.

Secondly, the reliance on data collected at a single point in time, may hinder the ability to draw definitive conclusions about cause-and-effect relationships. The observed associations between the variables studied do not necessarily imply causation. To address this, subsequent investigations could adopt a longitudinal approach, allowing for a more in-depth analysis over time.

Thirdly, the potential issue arises from the methodology of gathering information from individual participants for all variables. This approach may introduce common method bias. Nevertheless, several distinct techniques were employed to evaluate this potential bias, and the results suggest that it may not significantly impact the study's validity.

Lastly, it is important to acknowledge that the study employed a convenience sampling method, which inherently limits the representativeness of the sample. The sample was primarily drawn from construction projects in a single nation, which raises questions about the broader generalization of the findings. To overcome this, future research endeavors should consider expanding data collection to encompass multiple countries and industries. This approach would enhance the robustness of the findings across diverse settings.

## 7 Conclusion

This study investigated the intricate relationship between transformational leadership and project success in the context of Chinese construction projects, with a particular focus on the mediating roles of team reflexivity and project team resilience. The results show that both team reflexivity and project team resilience serve as mediators in the relationship between transformational leadership and project success. This study contributes to the existing body of knowledge by highlighting the potential mechanisms through which transformational leadership influences project outcomes. The results underline the importance of fostering team adaptation processes within project teams, particularly reflexivity and resilience, as possible factors in translating effective leadership into project success. By emphasizing the development of transformational leadership and the nurturing of team adaptation processes, project-based organizations may enhance their potential for achieving project success in the dynamic and challenging environment of the construction industry. Future research could explore longitudinal studies or qualitative approaches to validate these findings in different contexts, providing deeper insights into how transformational leadership and team adaptation processes evolve over time and across diverse project settings.

## Data Availability

The original contributions presented in the study are included in the article/Supplementary material, further inquiries can be directed to the corresponding author.

## References

[B1] Aga D. A. Noorderhaven N. Vallejo B. (2016). Transformational leadership and project success: the mediating role of team-building. Int. J. Proj. Manag. 34, 806–818. 10.1016/j.ijproman.2016.02.01236186280

[B2] Ahmad M. K. Abdulhamid A. B. Wahab S. A. Nazir M. U. (2022). Impact of the project manager's transformational leadership, influenced by mediation of self-leadership and moderation of empowerment, on project success. Int. J. Manag. Proj. Bus. 15, 842–864. 10.1108/IJMPB-03-2021-0066

[B3] Akinosho T. D. Oyedele L. O. Bilal M. Ajayi A. O. Delgado M. D. Akinade O. O.. (2020). Deep learning in the construction industry: a review of present status and future innovations. J. Build. Eng. 32, 101827. 10.1016/j.jobe.2020.101827

[B4] Al-edenat M. (2018). Reinforcing innovation through transformational leadership: mediating role of job satisfaction. J. Organ. Change Manag. 31, 810–838. 10.1108/JOCM-05-2017-0181

[B5] Ali H. Chuanmin S. Ahmed M. Mahmood A. Khayyam M. Tikhomirova A. (2021). Transformational leadership and project success: serial mediation of team-building and teamwork. Front. Psychol. 12, 689311. 10.3389/fpsyg.2021.689311 34557131 PMC8453157

[B6] Alliger G. M. Cerasoli C. P. Tannenbaum S. I. Vessey W. B. (2015). Team resilience: how teams flourish under pressure. Organ. Dyn. 44, 176–184. 10.1016/j.orgdyn.2015.05.003

[B7] Alwali J. Alwali W. (2022). The relationship between emotional intelligence, transformational leadership, and performance: a test of the mediating role of job satisfaction. Leadersh. Organ. Dev. J. 43, 928–952. 10.1108/LODJ-10-2021-0486

[B8] Antonakis J. House R. J. (2013). “The Full-Range Leadership Theory: The Way Forward,” in Transformational and Charismatic Leadership: The Road Ahead 10th Anniversary Edition, (Bingley: Emerald Group Publishing Limited), 3–33.

[B9] Arnold K. A. (2017). Transformational leadership and employee psychological well-being: A review and directions for future research. J. Occup. Health Psychol. 22, 381–393. 10.1037/ocp0000062 28150998

[B10] Atkinson R. (1999). Project management: cost, time and quality, two best guesses and a phenomenon, its time to accept other success criteria. Int. J. Proj. Manag. 17, 337–342. 10.1016/S0263-7863(98)00069-6

[B11] Avolio B. J. Bass B. M. Jung D. I. (1999). Re-examining the components of transformational and transactional leadership using the multifactor leadership. J. Occup. Organ. Psychol. 72, 441–462. 10.1348/096317999166789

[B12] Avolio B. J. Zhu W. Koh W. Bhatia P. (2004). Transformational leadership and organizational commitment: mediating role of psychological empowerment and moderating role of structural distance. J. Organ. Behav. 25, 951–968. 10.1002/job.283

[B13] Bandura A. (1999). Social cognitive theory: an agentic perspective. Asian J. Soc. Psychol. 2, 21–41. 10.1111/1467-839X.00024 11148297

[B14] Bass B. M. (1999). Two decades of research and development in transformational leadership. Eur. J. Work Organ. Psychol. 8, 9–32. 10.1080/135943299398410

[B15] Bell B. S. Kozlowski S. W. J. (2002). A typology of virtual teams: implications for effective leadership. Group Organ. Manag. 27, 14–49. 10.1177/1059601102027001003

[B16] Bush T. (2018). Transformational leadership: exploring common conceptions. Educ. Manag. Adm. Leadersh. 46, 883–887. 10.1177/1741143218795731

[B17] Carless S. A. Wearing A. J. Mann L. (2000). A short measure of transformational leadership. J. Bus. Psychol. 14, 389–405. 10.1023/A:1022991115523

[B18] Chai D. S. Hwang S. J. Joo B.-K. (2017). Transformational leadership and organizational commitment in teams: the mediating roles of shared vision and team-goal commitment: leadership and commitment in teams. Perform. Improv. Q. 30, 137–158. 10.1002/piq.21244

[B19] Crawford L. French E. Lloyd-Walker B. (2013). From outpost to outback: project career paths in Australia. Int. J. Proj. Manag. 31, 1175–1187. 10.1016/j.ijproman.2013.03.003

[B20] Crede M. Jong J. Harms P. (2019). The generalizability of transformational leadership across cultures: a meta-analysis. J. Manag. Psychol. 34, 139–155. 10.1108/JMP-11-2018-050639688105

[B21] Dogan I. Özdamar K. (2017). The effect of different data structures, sample sizes on model fit measures. Commun. Stat. - Simul. Comput. 46, 7525–7533. 10.1080/03610918.2016.1241409

[B22] Edmondson A. (1999). Psychological safety and learning behavior in work teams. Adm. Sci. Q. 44, 350–383. 10.2307/2666999

[B23] Elbanna S. (2015). Intuition in project management and missing links: analyzing the predicating effects of environment and the mediating role of reflexivity. Int. J. Proj. Manag. 33, 1236–1248. 10.1016/j.ijproman.2015.02.004

[B24] Fareed M. Z. Su Q. (2022). Transformational leadership and project success: a mediating role of public service motivation. Adm. Soc. 54, 690–713. 10.1177/00953997211040466 36186280 PMC9520658

[B25] Fareed M. Z. Su Q. Almutairi M. Munir K. Fareed M. M. S. (2022). Transformational leadership and project success: the mediating role of trust and job satisfaction. Front. Psychol. 13. 10.3389/fpsyg.2022.954052 36186280 PMC9520658

[B26] Fareed M. Z. Su Q. Aslam M. U. (2023). Transformational leadership and project success: the mediating role of psychological empowerment. SAGE Open 13, 21582440231154796. 10.1177/2158244023115479636186280

[B27] Giezen M. Bertolini L. Salet W. (2015). Adaptive capacity within a mega project: a case study on planning and decision-making in the face of complexity. Eur. Plan. Stud. 23, 999–1018. 10.1080/09654313.2014.916254

[B28] Guo H. Su Z. Ahlstrom D. (2016). Business model innovation: the effects of exploratory orientation, opportunity recognition, and entrepreneurial bricolage in an emerging economy. Asia Pac. J. Manag. 33, 533–549. 10.1007/s10490-015-9428-x

[B29] Han H. Ma F. Liu X. (2024). Transformational leadership and project success: the serial meditating roles of team flexibility and team agility. Front. Built Environ. 9. 10.3389/fbuil.2023.1334413

[B30] Hoch J. E. Bommer W. H. Dulebohn J. H. Wu D. (2018). Do ethical, authentic, and servant leadership explain variance above and beyond transformational leadership? A meta-analysis. J. Manag. 44, 501–529. 10.1177/0149206316665461

[B31] Hoegl M. Parboteeah K. P. (2006). Team reflexivity in innovative projects. R Manag. 36, 113–125. 10.1111/j.1467-9310.2006.00420.x

[B32] Ika L. A. (2009). Project success as a topic in project management journals. Proj. Manag. J. 40, 6–19. 10.1002/pmj.20137

[B33] Ika L. A. Donnelly J. (2017). Success conditions for international development capacity building projects. Int. J. Proj. Manag. 35, 44–63. 10.1016/j.ijproman.2016.10.005

[B34] Jiang S. Ling F. Y. Y. Ma G. (2024). Fostering resilience in project teams: adaptive structuration perspective. J. Manag. Eng. 40:04023047. 10.1061/JMENEA.MEENG-561527357964

[B35] Jugdev K. Müller R. (2005). A retrospective look at our evolving understanding of project success. Proj. Manag. J. 36, 19–31. 10.1177/875697280503600403

[B36] Kahai S. Jestire R. Huang R. (2013). Effects of transformational and transactional leadership on cognitive effort and outcomes during collaborative learning within a virtual world. Br. J. Educ. Technol. 44, 969–985. 10.1111/bjet.12105

[B37] Khan A. N. Khan N. A. (2022). The nexuses between transformational leadership and employee green organisational citizenship behaviour: role of environmental attitude and green dedication. Bus. Strategy Environ. 31, 921–933. 10.1002/bse.2926

[B38] Kloppenborg T. J. Petrick J. A. (1999). Leadership in project life cycle and team character development. Proj. Manag. J. 30, 8–13. 10.1177/875697289903000203

[B39] Kloutsiniotis P. V. Mihail D. M. Mylonas N. Pateli A. (2022). Transformational leadership, HRM practices and burnout during the COVID-19 pandemic: the role of personal stress, anxiety, and workplace loneliness. Int. J. Hosp. Manag. 102, 103177. 10.1016/j.ijhm.2022.103177 35079194 PMC8776468

[B40] Le P. B. Lei H. (2019). Determinants of innovation capability: the roles of transformational leadership, knowledge sharing and perceived organizational support. J. Knowl. Manag. 23, 527–547. 10.1108/JKM-09-2018-0568

[B41] Lei X. H. Liu W. Su T. Y. Shan Z. W. (2022). Humble leadership and team innovation: the mediating role of team reflexivity and the moderating role of expertise diversity in teams. Front. Psychol. 13, 726708. 10.3389/fpsyg.2022.726708 35572304 PMC9097902

[B42] Lin C.-P. Liu C.-M. Liao W.-S. (2020). Being excellent: predicting team performance based on social cognitive theory and social identification theory. Total Qual. Manag. Bus. Excell. 10.1080/14783363.2018.1485483

[B43] Liu X. Kassa A. Tekleab A. G. (2025). Are intrateam trust and organizational trust substitutable? Effects on team reflexivity, engagement and performance. J. Bus. Res. 189:115164. 10.1016/j.jbusres.2024.115164

[B44] Locke E. A. Latham G. P. (2002). Building a practically useful theory of goal setting and task motivation: a 35-year odyssey. Am. Psychol. 57, 705–717. 10.1037/0003-066X.57.9.705 12237980

[B45] Lu H. Li F. (2021). The dual effect of transformational leadership on individual- and team-level performance: the mediational roles of motivational processes. Front. Psychol. 12, 606066. 10.3389/fpsyg.2021.606066 33815201 PMC8009998

[B46] Marks M. A. Mathieu J. E. Zaccaro S. J. (2001). A temporally based framework and taxonomy of team processes. Acad. Manage. Rev. 26, 356–376. 10.5465/amr.2001.4845785

[B47] Meneghel I. Salanova M. Martínez I. M. (2016). Feeling good makes us stronger: how team resilience mediates the effect of positive emotions on team performance. J. Happiness Stud. 17, 239–255. 10.1007/s10902-014-9592-6

[B48] Mokhber M. Khairuzzaman W. Vakilbashi A. (2018). Leadership and innovation: the moderator role of organization support for innovative behaviors. J. Manag. Organ. 24, 108–128. 10.1017/jmo.2017.26

[B49] Pavez I. Gómez H. Laulié L. González V. A. (2021). Project team resilience: the effect of group potency and interpersonal trust. Int. J. Proj. Manag. 39, 697–708. 10.1016/j.ijproman.2021.06.004

[B50] Pieterse A. N. van Knippenberg D. van Ginkel W. P. (2011). Diversity in goal orientation, team reflexivity, and team performance. Organ. Behav. Hum. Decis. Process. 114, 153–164. 10.1016/j.obhdp.2010.11.003

[B51] Pinto J. K. Mantel S. J. (1990). The causes of project failure. IEEE Trans. Eng. Manag. 37, 269–276. 10.1109/17.62322

[B52] Podsakoff P. M. MacKenzie S. B. Lee J.-Y. Podsakoff N. P. (2003). Common method biases in behavioral research: A critical review of the literature and recommended remedies. J. Appl. Psychol. 88, 879–903. 10.1037/0021-9010.88.5.879 14516251

[B53] Pollack J. Helm J. Adler D. (2018). What is the iron triangle, and how has it changed? Int. J. Manag. Proj. Bus. 11, 527–547. 10.1108/IJMPB-09-2017-0107

[B54] Raziq M. M. Borini F. M. Malik O. F. Ahmad M. Shabaz M. (2018). Leadership styles, goal clarity, and project success: evidence from project-based organizations in Pakistan. Leadersh. Organ. Dev. J. 39, 309–323. 10.1108/LODJ-07-2017-0212

[B55] Ren S. Wang Z. Collins N. T. (2021). The joint impact of servant leadership and team-based HRM practices on team expediency: the mediating role of team reflexivity. Pers. Rev. 50, 1757–1773. 10.1108/PR-07-2020-0506

[B56] Sahin N. Bilir F. P. (2024). The effect of transformational leadership and personal cultural values on creating a learning organization. Humanit. Soc. Sci. Commun. 11, 1–9. 10.1057/s41599-024-02701-623522804

[B57] Sanchez-Manzanares M. Rico R. Antino M. Uitdewilligen S. (2020). The joint effects of leadership style and magnitude of the disruption on team adaptation: a longitudinal experiment. Group Organ. Manag. 45, 836–864. 10.1177/1059601120958838

[B58] Schippers M. C. Den Hartog D. N. Koopman P. L. Van Knippenberg D. (2008). The role of transformational leadership in enhancing team reflexivity. Hum. Relat. 61, 1593–1616. 10.1177/0018726708096639

[B59] Schippers M. C. West M. A. Dawson J. F. (2015). Team reflexivity and innovation: the moderating role of team context. J. Manag. 41, 769–788. 10.1177/0149206312441210

[B60] Scott-Young C. Samson D. (2008). Project success and project team management: Evidence from capital projects in the process industries. J. Oper. Manag. 26, 749–766. 10.1016/j.jom.2007.10.006

[B61] Shaikh F. Afshan G. Anwar R. S. Abbas Z. Chana K. A. (2023). Analyzing the impact of artificial intelligence on employee productivity: the mediating effect of knowledge sharing and well-being. Asia Pac. J. Hum. Resour. 61, 794–820. 10.1111/1744-7941.12385

[B62] Stoverink A. C. Kirkman B. L. Mistry S. Rosen B. (2020). Bouncing back together: toward a theoretical model of work team resilience. Acad. Manage. Rev. 10.5465/amr.2017.0005

[B63] Triandis H. C. (2001). Individualism-collectivism and personality. J. Pers. 69, 907–924. 10.1111/1467-6494.696169 11767823

[B64] Varajão J. Fernandes G. Amaral A. Gonçalves A. M. (2021). Team resilience model: an empirical examination of information systems projects. Reliab. Eng. Syst. Saf. 206, 107303. 10.1016/j.ress.2020.107303

[B65] Vaske J. J. Beaman J. Sponarski C. C. (2017). Rethinking internal consistency in cronbach's alpha. Leis. Sci. 39, 163–173. 10.1080/01490400.2015.1127189

[B66] Wang H.-J. Demerouti E. Le Blanc P. (2017). Transformational leadership, adaptability, and job crafting: The moderating role of organizational identification. J. Vocat. Behav. 100, 185–195. 10.1016/j.jvb.2017.03.009

[B67] Wang Z. Liang Q. Yan Z. Liu J. Liu M. Wang X.. (2023). The association between team resilience and team performance in nurses during COVID-19 pandemic: a network analysis. BMC Nurs. 22, 54. 10.1186/s12912-023-01216-w 36841817 PMC9959955

[B68] Wang Z. Ren S. Chadee D. Liu M. Cai S. (2021). Team reflexivity and employee innovative behavior: the mediating role of knowledge sharing and moderating role of leadership. J. Knowl. Manag. 25, 1619–1639. 10.1108/JKM-09-2020-0683

[B69] Watson C. B. Chemers M. M. Preiser N. (2001). Collective efficacy: a multilevel analysis. Pers. Soc. Psychol. Bull. 27, 1057–1068. 10.1177/0146167201278012

[B70] West M. A. Anderson N. R. (1996). Innovation in top management teams. J. Appl. Psychol. 81, 680–693. 10.1037/0021-9010.81.6.680

[B71] Wood R. Bandura A. (1989). Social cognitive theory of organizational management. Acad. Manage. Rev. 14, 361–384. 10.5465/amr.1989.4279067

[B72] Zaman U. Nawaz S. Tariq S. Humayoun A. A. (2019). Linking transformational leadership and “multi-dimensions” of project success: Moderating effects of project flexibility and project visibility using PLS-SEM. Int. J. Manag. Proj. Bus. 13, 103–127. 10.1108/IJMPB-10-2018-0210

[B73] Zhu W. Newman A. Miao Q. Hooke A. (2013). Revisiting the mediating role of trust in transformational leadership effects: do different types of trust make a difference? Leadersh. Q. 24, 94–105. 10.1016/j.leaqua.2012.08.004

