# Supplementary material for: Transformational leadership and project success: the mediating roles of team reflexivity and project team resilience
Source: Front Psychol. 2025 Apr 30;16:1504108. doi: 10.3389/fpsyg.2025.1504108 (PMC12075218; doi:10.3389/fpsyg.2025.1504108)
Supplement: Supplementary file 1 [file Supplementary_file_1.docx]

Transformational Leadership and Project Success: the Mediating Roles of Team Reflexivity and Project Team Resilience

Huibin Han^1*^, Chihao Ma^1^, Danning Yang^2^, Weiwei Zhao^2^

^1^School of Economics and Management, Liaoning University of Technology, Jinzhou 121000, Liaoning Province, China

^2^Liaoning Institute of Science and Engineering, Jinzhou 121000, Liaoning Province, China

*** Correspondence:**Huibin Han^*^
hanhuibin123@lnut.edu.cn

Keywords: transformational leadership_1_, team reflexivity_2_, project team resilience_3_, project success_4_, project management_5_.

# Appendix

# Survey questionnaires

# Transformational leadership

My project manager communicates a clear and positive vision of the future.

My project manager treats staff as individuals, supports and encourages their development.

My project manager gives encouragement and recognition to staff.

My project manager fosters trust, involvement and cooperation among team members.

My project manager encourages thinking about problems in new ways and questions assumptions.

My project manager is clear about his/her values and practices what he/she preaches.

My project manager instills pride and respect in others and inspires me by being highly competent.

# Team reflexivity

Our team investigated and observed the context and the progress of the project (e.g., task performance strategies, goals, project requirements, the organizational context, etc.).

Our team adjusted its task performance strategies in response to changes in the context and progress of the project.

Our team spent an adequate amount of time considering the likely consequences of its task activities (e.g. considerations regarding usefulness of the project, compatibility with other projects, cost, etc.).

Strategies and work approaches chosen were later checked for their appropriateness by our project team.

# Project team resilience

In my team, we cope well with the conflicts we experience at work.

In my team, we cope well with the tensions we experience at work.

In my team, we cope well with the pressures we experience at work.

In my team, even during times of stress and pressure, we always manage to find effective solutions.

# Project success

The project was completed on time.

The project was completed according to the budget allocated.

The outcomes of the project are used by its intended end users.

The outcomes of the project are likely to be sustained.

The outcomes of the project have directly benefited the intended end users, either through increasing efficiency or effectiveness.

Given the problem for which it was developed, the project seems to do the best job of solving that problem.

Project team members were satisfied with the process by which the project was implemented.

The project had no or minimal start-up problems because it was readily accepted by its end users.

Project specifications were met by the time of handover to the target beneficiaries.
